# CTP synthase 2 predicts inferior survival and mediates DNA damage response via interacting with BRCA1 in chronic lymphocytic leukemia

**DOI:** 10.1186/s40164-022-00364-0

**Published:** 2023-01-12

**Authors:** Xinting Hu, Yang Han, Jiarui Liu, Hua Wang, Zheng Tian, Xin Zhang, Ya Zhang, Xin Wang

**Affiliations:** 1 Department of Hematology, Shandong Provincial Hospital, Shandong University, No.324, Jingwu Road, Jinan, 250021 Shandong China; 2 Department of Hematology, Shandong Provincial Hospital Affiliated to Shandong First Medical University, No.324, Jingwu Road, Jinan, 250021 Shandong China; 3 Shandong Provincial Engineering Research Center of Lymphoma, Jinan, 250021 Shandong China; 4 Branch of National Clinical Research Center for Hematologic Diseases, Jinan, 250021 Shandong China; 5 National Clinical Research Center for Hematologic Diseases, The First Affiliated Hospital of Soochow University, Suzhou, 251006 China

**Keywords:** Chronic lymphocytic leukemia, CTP synthase 2, Survival, DNA damage response, Breast cancer associated 1

## Abstract

**Background:**

Cytidine triphosphate synthase 2 (CTPS2) is an essential metabolic enzyme that catalyzes the biosynthesis of CTP. CTP synthases contribute to lymphocytes proliferation and tumorigenesis, but the role of CTPS2 in chronic lymphocytic leukemia (CLL) remains undefined.

**Methods:**

In silico analysis was performed to quantified the expression and clinical analysis of CTPS2 and BRCA1. The expression was then validated on the internal sets. Loss-and gain-of-function assays were conducted to investigate the physiological phenotypes in CLL. RNA-seq was employed to probe the molecular mechanism of CTPS2.

**Results:**

Herein, significant elevated expression of CTPS2 was observed in CLL patients compared to normal CD19 + B cells, which was verified in three independent cohorts. Furthermore, overexpression of CTPS2 was closely associated with undesired prognostic indicators, including unmutated IGHV status and chromosome 11q23 deletion. Additionally, elevated CTPS2 expression predicted adverse overall survival and treatment-free survival with independent prognostic significance. Downregulation of CTPS2 in CLL cells exhibited attenuated cell proliferation, arrested G2/M cell cycle and increased apoptosis. The addition of CTP or glutamine could reverse the above effects. Since RNA-seq showed the enrichment in DNA damage and response signaling, we subsequently found that silence of CTPS2 remarkably elevated DNA damage and decreased DNA repair. It was demonstrated that CTPS2 mediated DNA damage response via interacting with Breast Cancer 1 (BRCA1) protein in CLL through CoIP assays and rescued experiments.

**Conclusions:**

Collectively, our study generated the novel findings that CTPS2 promoted CLL progression via DNA damage response and repair pathway. Targeting nucleotide metabolism potentially became an attractive strategy for treatment against CLL.

**Supplementary Information:**

The online version contains supplementary material available at 10.1186/s40164-022-00364-0.

## Background

Chronic lymphocytic leukemia (CLL) is the most common form of adult leukemia in Europe, Australia, and America [1, 2]. The past decades have witnessed a massive revolution in targeted therapy of chronic lymphocytic leukemia, with the approval of novel agents, including small molecule inhibitors targeting Bruton’s tyrosine kinase, phosphatidylinositol-3-kinase, and B-cell lymphoma 2 [3–5]. However, side effects and drug resistance of these agents are still difficult to avoid due in part to off-target effects [6–8]. Further insights into the novel targets may therefore reveal essential pathophysiological clues that can lead to more individualized approaches to achieve durable disease control in the majority of patients in the future.

As the isozyme of cytidine triphosphate synthase (CTPS), it has been reported that Epstein-Barr virus could upregulate CTPS2 to meet CTP demand in newly EBV-infected B cells and to drive the pathogenesis of lymphoma [9]. CTPS is the key regulatory enzyme in pyrimidine biosynthesis, with critical roles in the regulation of energy metabolism as well as the biosynthesis of nucleic acids, phospholipids and membranes [10, 11]. CTPS catalyzes one of the key regulatory and rate-limiting steps of CTP synthesis by converting the uridine triphosphate to cytidine triphosphate [12, 13]. While, glutamine (Gln) is an essential source of nitrogen atoms at the process of this CTP biosynthesis [14]. Recently, there has been increasing evidence that CTPS is frequently mis-regulated in cancers [15, 16]. There are two CTPS isoforms encoded on separate genes, CTPS1 and CTPS2, which share 75% identity. However, their relative roles remain unclear. Previous study has suggested that CTPS1 deletion could cause severe immune deficiency due to serious defects in lymphocyte proliferation [17, 18]. The function and clinical significance of CTPS2 in tumorigenesis remains an open question.

Yet, the role of CTPS2 had not been explored in chronic lymphocytic leukemia. Hence, this study was aimed to investigate the role of CTPS2 in CLL pathogenesis and the mechanisms that underlie the association between CTPS2 and malignancy. In the work presented here, we provided compelling data that CTPS2 not only enhanced proliferation of CLL but also interacted with BRCA1 and there by impacted DNA damage response (DDR) in CLL. These data opened up new perspectives on key pathophysiological mechanisms that could be exploited for biomarker development to guide treatment choices in CLL.

## Methods

### Patient and sample characteristics

Blood samples of 96 de novo patients of CLL were collected at the Department of Hematology in Shandong Provincial Hospital after informed consent as approved by the ethics commission. The leukemic clone proportion in peripheral blood of the enrolled CLL patients was > 90%, which could be represented by peripheral blood mononuclear cells [19]. All the participants were aged between 29 and 85 years (mean age = 60.29 years, s. d. = 11.45). Peripheral blood mononuclear cells (PBMCs) were isolated by Ficoll density gradient centrifugation, which was described in previous study [20, 21]. CD19 + B cells from healthy donors were purified using CD19 + magnetic microbeads kit (Miltenyi Biotec, Bergisch Gladbach, Germany). Clinical characteristics of CLL patients, including age, stage, immunoglobulin mutational status, and cytogenetics (FISH) were recorded.

### In silico* analysis*

1030 clinically annotated CLL patients from multiple cohorts were enrolled in this study. Normalized microarray and RNA-seq data from the GSE50006, GSE22529, GSE55288, GSE22762, GSE39671 datasets were downloaded from the Gene Expression Omnibus (GEO) database. For the ICGC cohort, gene expression and clinical data were obtained from the International Cancer Genome Consortium database (https://icgc.org/). The cut-off value was assessed based on median mRNA expression level. The Kaplan‒Meier method was used for survival analysis.

### Cell culture and reagents

Human CLL cell lines were routinely cultured in IMDM (MEC-1) and RPMI-1640 (EHEB) medium with 10% fetal bovine serum (Gibco, MD, USA). The medium contained a 1% penicillin/streptomycin mixture. Cells were incubated at 37 °C in humidified air containing 5% CO2. Complete medium without Gln was purchased from M&C GENE (Beijing, China). Cytidine-triphosphate (HY-125818, MCE) were soluble in sterile saline solution to the storage concentration at 10 mM.

### Lentivirus and plasmids mediated regulation of CTPS2 and BRCA1

The sequences for CTPS2 shRNAs were as follows: shCTPS2#1, 5′-CCGAGGACCCTGTGAAATT-3′; shCTPS2#2,5′-GCAGTGATAGAGTTTGCAA -3′; shCTPS2#3, 5′-CCACAGAGTTTAGGCCAAA-3′. The shRNAs for CTPS2, lentivirus-BRCA1 and the negative control RNA (shControl) were synthesized and purified by OBIO (Shanghai, China). The shRNAs were cloned into lentiviral vectors and lentivirus infection was carried out according to the manufacturer’s instruction. Forty-eight hours after transfection, stably silenced clones were selected by 2 μg/ml puromycin. The medium was changed at twenty-four hours after transfection, and cells were harvested for subsequent analysis seventy-two hours after transfection. Plasmids delivery to overexpress CTPS2 was constructed by WZ Biosciences (Jinan, Shandong, China).

### RNA isolation and quantitative real-time PCR

Total RNA was purified with Trizol reagent (Invitrogen, MA, USA) and reversed transcribed into cDNA using a reverse transcription kit (TaKaRa, Dalian, China). Real-time PCR was performed in a Light Cycler 480 II Real-Time PCR system (Roche Diagnostics, Basel, Switzerland) using TB Green (Takara). Real-time PCR of each sample was performed in triplicate. Results were obtained using the sequence detection software Light cycler 480 and analyzed using GraphPad Prism version 7.0 statistical software.

### Western blot analysis

Whole cell lysates were extracted using radioimmunoprecipitation assay (RIPA) buffer together with a 1 × phosphatase inhibitor cocktail (PhosSTOP, Roche, Basel, Switzerland). The primary antibodies used were as follows: anti-CTPS2 (ab196016, Abcam, Cambridge, UK), anti-cyclin B1 (ab32053,Abcam), anti-CDK1 (ab18, Abcam), anti-phospho-H2AX (7631, Cell Signaling Technology, MA,USA), anti-phospho-ATM (13050, Cell Signaling Technology), anti-phospho-BRCA1 (14823, Cell Signaling Technology), anti-Bcl-2 (4223, Cell Signaling Technology), anti-Bax (14796, Cell Signaling Technology), anti-cleaved PARP (5625, Cell Signaling Technology), anti-p21 (2947, Cell Signaling Technology), β-actin (TA-08, Zhongshan Goldenbridge), and anti-GAPDH (TA-09, Zhongshan Goldenbridge, Beijing, China).

### Cell proliferation assessment

Cell viability was assayed using the Cell Counting Kit-8 (CCK-8) (Dojindo, Japan) as previously described. Briefly, the cell density was adjusted to 1 × 10^4^ cells/100 μl/well and inoculated in 96-well plates. Thereafter, CCK-8 reagents were added to each well and incubated for 3 h. Finally, optical density values were read using the SpectraMax M2 Microplate Reader (Molecular Devices, CA, USA) at 450 nm to ascertain cell proliferation capability.

### Analyses of cell apoptosis and cell cycle

The analyses of the cell cycle distributions and the measurements of the percentage of apoptotic cells were performed by flow cytometry using Navios flow cytometer (Beckman Coulter, CA, USA). For cell cycle analysis, cells were washed with PBS, fixed with cold 70% alcohol at-20 °C overnight before stained with PI (BD Biosciences, MA, USA). The apoptosis detection was performed by using the Annexin V-PE/7AAD apoptosis detection kit and according to manufacturer’s instructions (BD Biosciences). The percentage of cells in the indicated cell cycle phase was calculated with ModFit LT software.

### RNA-sequencing

RNA was exacted using the Trizol reagent (Invitrogen, MA, USA). RNA-seq experiments were performed by Novogene (Beijing, China). After reverse transcription and cDNA library enrichment, six RNA-sequencing (RNA-seq) libraries (three for shCTPS2 group and three for shControl group) were generated following the manufacturer’s recommendations. Purified library DNA was captured on an Illumina flowcell for cluster generation and sequenced on an Illumina HiSeq platform, and the fragments per kilobase of transcript per million fragments mapped (FPKM) of each gene were calculated. Differentially expressed genes were determined by the DESeq2 Bioconductor/R package (https://doi.org/10.18129/B9.bioc.DESeq2). The visualization of Gene Ontology (GO) and Kyoto Encyclopedia of Genes and Genomes (KEGG) analyses were implemented using the R package ClusterProfiler (v3.0.0). Gene counts for heatmap generation were converted into z-scores and input into the ComplexHeatmap Bioconductor/R package [22].

### Immunofluorescence

Cells were fixed with 4% paraformaldehyde/PBS for 15 min and permeabilized with 0.5% Triton X-100 (in 1 × PBS) for 20 min at room temperature. Primary antibodies for staining CTPS2 (ab32087, Abcam), BRCA1 (Santa Cruz, TX, USA) and p-H2AX (ab81299, Abcam) were used at 1:200 for 1 h at room temperature. After three washes with PBS, the sections were incubated with the secondary antibody in PBS with 2% normal serum for 1 h at room temperature, followed by three washes in PBST. Subsequently, cells were stained with 4, 6-diamidino-2-phenylindole dihydrochloride (DAPI; a DNA-specific fluorescent dye) for 5–10 min. Stained cells were covered with coverslips and visualized using confocal microscope.

### Comet assay

Alkaline single-cell gel electrophoresis assay was performed according to the protocol from Trevigen (4250–050-K). Briefly, cells were plated on CometSlides after dilution in low melting point agarose. Lysis solution overnight allowed for electrophoretic separation of DNA fragments and DAPI was used to stain the DNA fragments. At least 50 cells per treatment group were imaged and images were analyzed with Comet Assay IV Software Version 4.3 to calculate the tail moment.

### Statistical analysis

The data were expressed as mean values ± standard error of mean (SEM) from at least three independent experiments. The significance of differences between groups was analyzed using Students t-tests and one-way analysis of variance (ANOVA). The overall survival (OS) time was calculated from the date of diagnosis to the date of death or the last follow-up date. The Kaplan–Meier method and log-rank tests were used for survival analysis. Cox proportional hazards regression models were used for univariate and multivariable analysis. All statistical analyses were performed with SPSS Statistics version 20.0 and GraphPad Prism version 7.0 statistical software. p < 0.05 was considered statistically significant.

## Results

### Clinical significance of CTPS2 expression in CLL

**Fig. 1:**
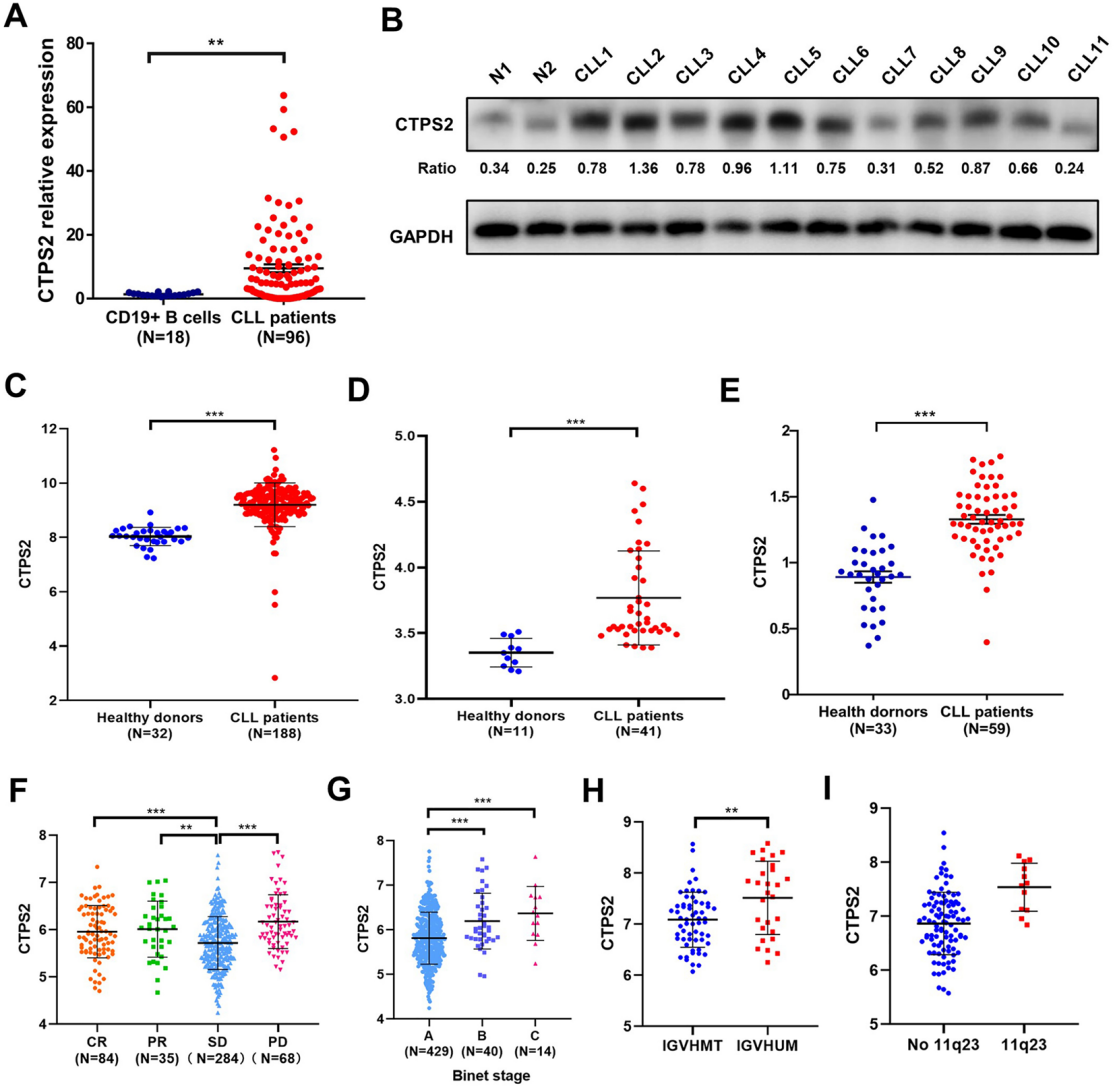
Aberrantly increased level of cytidine triphosphate synthase (CTPS2) expression in chronic lymphocytic leukemia (CLL). **A** The relative ratio of CTPS2 mRNA in CLL patients versus that in purified normal CD19 + B cells derived from healthy donors. **B** Protein expression of CTPS2 in CLL primary cells and healthy donors. **C**–**E** CTPS2 was markedly upregulated in CLL public database. Analyses were based on GSE5006, GSE22529 and GSE55288, respectively. **F** Expression of CTPS2 in stable disease (SD) and progressive disease (PD) patients was elevated relative to partial response (PR) and complete response (CR) patients. Data were from the ICGC database. **G**–**I** CLL patients with higher Binet stage, unmutated IGHV and 11q23^−^ presented high CTPS2 expression (ICGC database). Data were shown as the mean ± SEM. **p < 0.01, ***p < 0.001

To study the clinical relevance, the mRNA expression level of CTPS2 was examined in 96 CLL primary samples from Shandong Provincial Hospital CLL (SPHCLL) database. Quantitative real-time PCR confirmed the significant upregulation of CTPS2 in CLL patients compared to purified normal CD19 + B cells derived from healthy donors, with an over tenfold increase (Fig. 1A, *p* <0.01). CTPS2 expression was also confirmed to be higher in MEC-1 (1.68-fold change, *p* = 0.04) and EHEB cells (2.5- fold change, *p* < 0.001) than in normal B cells (Additional file 1: Fig. S1A). What’s more, the protein expression levels of CTPS2 in the CLL patients were increased significantly compared with those of the control group (Fig. 1B). Equivalent results could be obtained through bioinformatic analysis in another three independent cohorts (GSE5006, GSE22529 and GSE55288, Fig. 1C–E). Based on median expression level of mRNA, patients were divided into two groups: CTPS2^high^ and CTPS2^low^. Notably, the CTPS2^high^ group was significantly involved in undesirable prognostic indicators, including unfavorable therapeutic effect (Fig. 1F), high-risk Binet stage (Fig. 1G), IGHV un-mutation (Fig. 1H) and 11q23- (Fg. 1I) through analyzing ICGC database.

**Fig. 2:**
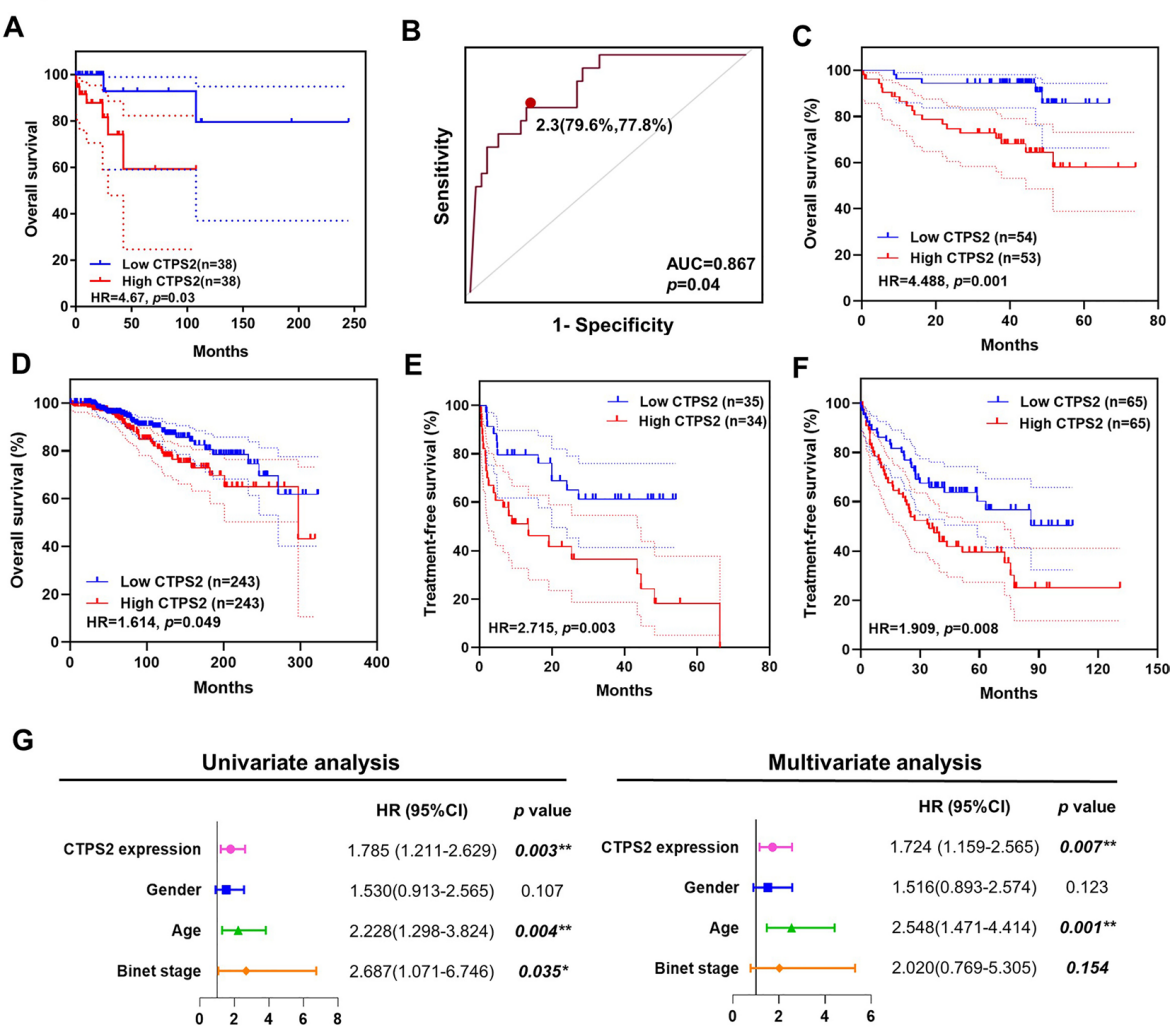
Clinical significance of CTPS2 expression in CLL. **A** Kaplan–Meier survival curves of CLL patients from Department of Hematology in Shandong Provincial Hospital CLL (SPHCLL) database. **B** ROC curves of CTPS2 level; AUC, area under the ROC curve. **C**, **D** The overall survival of CTPS2^high^ group was significantly shorter than CTPS2^low^ group. **E**, **F** The treatment-free survival of CTPS2^high^ group was significantly shorter than CTPS2.^low^ group. **G** Univariate and multivariate Cox analysis. Bold fonts indicate statistical significance. *p < 0.05; **p < 0.01

To explore the clinical and pathological role of CTPS2 in CLL, we analyzed the correlation of CTPS2 expression levels with clinical parameters. As Table 1 shown, high levels of CTPS2 expression were associated with the lack of IGHV mutation (*p* = 0.035) and unfavorable cytogenetics (*p* = 0.038), suggesting that upregulation of CTPS2 expression was associated with poor prognosis of CLL disease. Consistent with these data, Kaplan–Meier curves suggested a significant association between increased expression of CTPS2 and undesirable overall survival in CLL patients (Fig. 2A, *p* = 0.03). In the ROC curve analyses, the area under the ROC curve was 0.867, indicating a relatively accurate discrimination (Fig. 2B). In addition, it was validated that the CLL patients with CTPS2 overexpression had a significant decrease in overall survival both in GSE22762 (HR = 4.488, *p* = 0.001, Fig. 2C) and ICGC database (HR = 1.614, *p* = 0.049, Fig. 2D). Consistently, CTPS2 upregulation was also involved in unfavorable treatment-free survival in GSE22762 (HR = 2.715, *p* = 0.003, Fig. 2E) and GSE39671 cohorts (HR = 1.909, *p* = 0.008, Fig. 2F).

**Table 1:** Correlation between CTPS2 protein expression and clinicopathologic parameters of chronic lymphocytic leukemia (CLL) patients from Shandong Provincial Hospital CLL (SPHCLL) database

Characteristics	No	CTPS2 expression^a^		*p* value
		Low	High	
Age(years)				
< 60	33	16	17	0.994
≥ 60	35	17	18	
Gender				
Male	44	22	22	0.885
Female	29	14	15	
WBC Count (*10^9^/L)				
< 40	46	22	24	0.874
≥ 40	24	11	13	
Rai stage				
Low risk	42	19	23	0.218
High risk	14	4	10	
Binet stage				
Stage A or B	38	17	21	0.448
Stage C	15	5	10	
IGHV mutation				
Mutated	17	12	5	0.035*
Unmutated	6	1	5	
ZAP 70				
Positive	4	1	3	0.661
Negative	30	9	21	
CD38				
Positive	4	2	2	0.529
Negative	36	14	22	
Serum LDH				
Normal	50	22	28	0.384
Elevated	14	8	6	
Serum β2-MG				
Normal	36	15	21	0.581
Elevated	31	15	16	
17p				
Normal	36	14	22	0.528
Deletion	4	2	2	
FISH				
Favorable: normal,13q-, trisomy 12	23	10	13	0.038*
Unfavorable: 11q- and/ or 17p-	14	3	11	

Bold font indicates statistical significance

*WBC* White blood cell; *LDH* Lactate dehydrogenase; *β2-MG* β2-microglobulin; *HR* Hazard ratio; *CI* Confidence interval

^a^Cut-off value was based on median mRNA expression level of CTPS2

^*^p < 0.05

Furthermore, univariate Cox analysis implicated that the high expression of CTPS2 predicted adverse outcome in CLL patients (HR = 2.745, 95% CI 2.018–3.734, *p* < 0.001). Subsequently, the only two significant parameters, CTPS2 expression and β2-MG level, were further enrolled into multivariate analysis. The results of the multivariate Cox regression analysis suggested that CTPS2 was the independent prognostic indicator of overall survival (Table 2). Moreover, the same analysis was conducted in public databases, showing the independent prognostic value of CTPS2 in CLL (Fig. 2G).

**Table 2:** Cox regression analyses of overall survival (OS) in CLL patients

Variable		Univariate analysis			Multivariate analysis		
		HR	95% CI	*p* value	HR	95% CI	*p* value
Binet stage	C vs A/B	1.474	0.937–2.389	0.105			
CTPS2 expression	High vs low	2.745	2.018–3.734	0.001***	0.666	0.416–1.064	0.049*
β2-MG (mg/ L)	< 3 vs ≥ 3	0.021	0.003–0.126	0.001***	0.001	0.001–0.538	0.876
IGHV status	Unmutated vs mutated	2.923	0.000–87.321	0.844			
CD38	Positive vs negative	5.279	0.735–37.927	0.098			
ZAP70	Positive vs negative	21.715	0.109–36.449	0.254			

Bold font indicates statistical significance

*WBC* White blood cell; *LDH* Lactate dehydrogenase; *β2-MG* β2-microglobulin; *HR* Hazard ratio; *CI* Confidence interval

^*^p < 0.05

^***^p < 0.001

### CTPS2 mediated the cell growth, cell cycle arrest and apoptosis of CLL cells

**Fig. 3:**
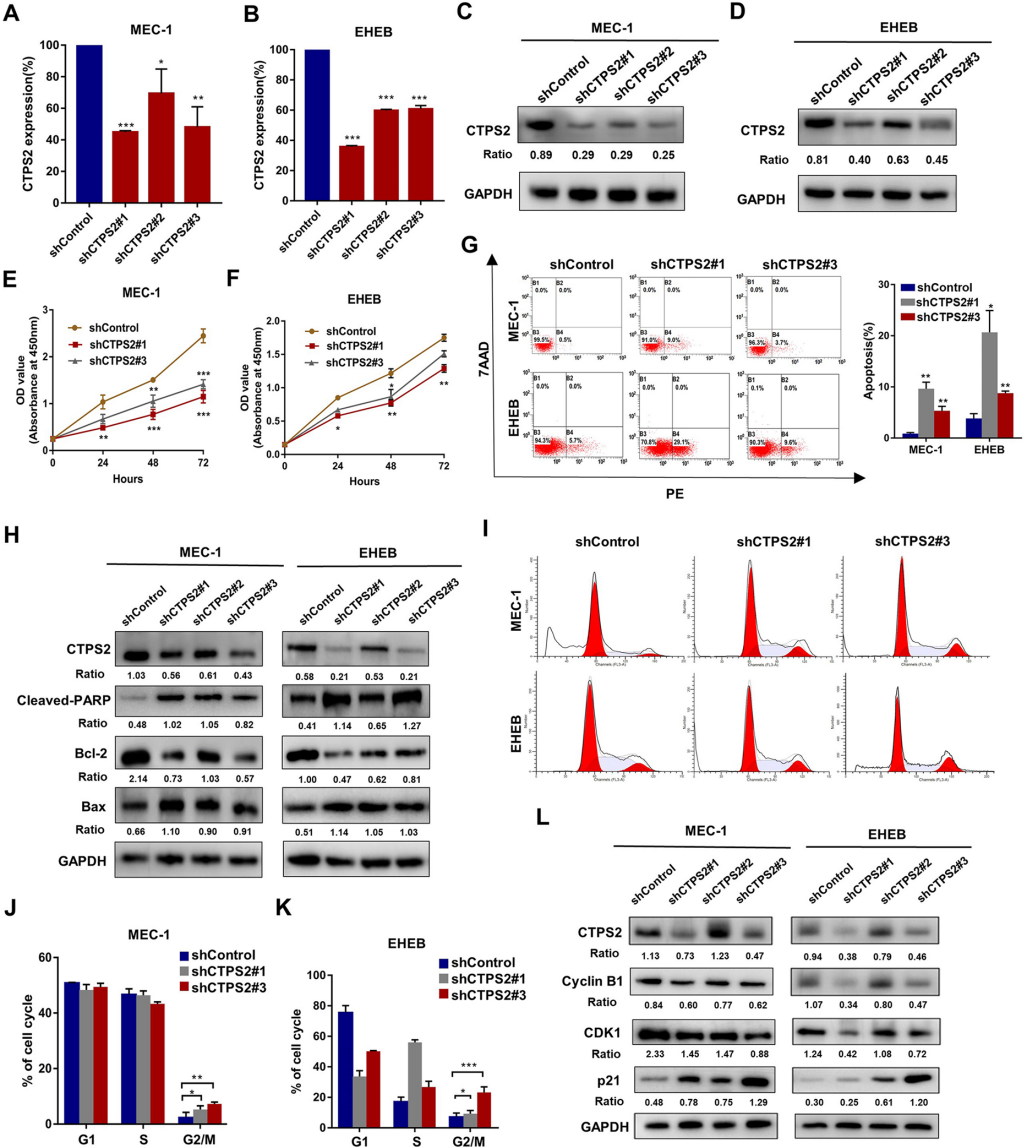
CTPS2 knockdown restrained the survival of CLL cell lines. **A**–**B** Real-time RT-PCR analysis of CTPS2 mRNA levels in control vs shCTPS2 in MEC-1 and EHEB cells. **C**, **D** Expression of the CTPS2 was checked by western blot (WB) after transfecting. **E**, **F** Proliferation curves of MEC-1 and EHEB cells transfected with shCTPS2 or shControl. **G** Representative dot plots generated by flow cytometry analysis of CTPS2-knockdown groups versus negative control. **H** CTPS2 interference induced enhanced apoptosis through western blot analysis. **I**–**K** Representative results for the cell cycle distributions of MEC-1 and EHEB cells with CTPS2 knockdown. **L** The protein expression levels of cyclin B1, CDK1, p21 and CTPS2 were detected in MEC-1 and EHEB cells. Data were shown as the mean ± SEM of at least three independent experiments. *p < 0.05, **p < 0.01, ***p < 0.001

Motivated by above finding, we sought to investigate the physiological phenotypes associated with CTPS2 in CLL. Three lentivirus-mediated RNA interference vectors targeting CTPS2 were used and two of them exhibited remarkable silence of CTPS2 at the mRNA and protein levels in MEC-1 (Fig. 3A, C) and EHEB cells (Fig. 3B, D). shCTPS2#1 demonstrated highest efficacy with 54% and 64% in MEC-1 and EHEB cells, respectively (*p* < 0.001). The CCK-8 assay was applied to assess the effect of CTPS2-loss on the proliferation of CLL cells. According to the results, stable transfection of shCTPS2 significantly suppressed CLL cell proliferation (Fig. 3E, F).

Subsequently, flow cytometry was performed to evaluate the apoptotic rates. CLL cells with CTPS2 knockdown showed obvious apoptotic rates relative to control transfected cells (Fig. 3G). Decreased expression levels of Bcl-2 and elevated levels of Bax as well as cleavage of the apoptotic marker PARP, were observed in CTPS2 knockdown cells (Fig. 3H). The cell cycle distribution was evaluated by flow cytometry, which demonstrated that silence of CTPS2 induced a blockade in cell cycle progression at the G2/M phase (Fig. 3I–K). As shown in Fig. 3L, the decreased levels of CDK1 and cyclin B1, checkpoint proteins of G2/M cell cycle, were observed. Concurrently, the protein p21 was increased. Therefore, the defection of CTPS2 restrained the growth of CLL cells predominantly through inhibition of cell apoptosis and induction of cell cycle arrest.

### CTPS2 promoted CLL by participating in Gln and CTP metabolism

**Fig. 4:**
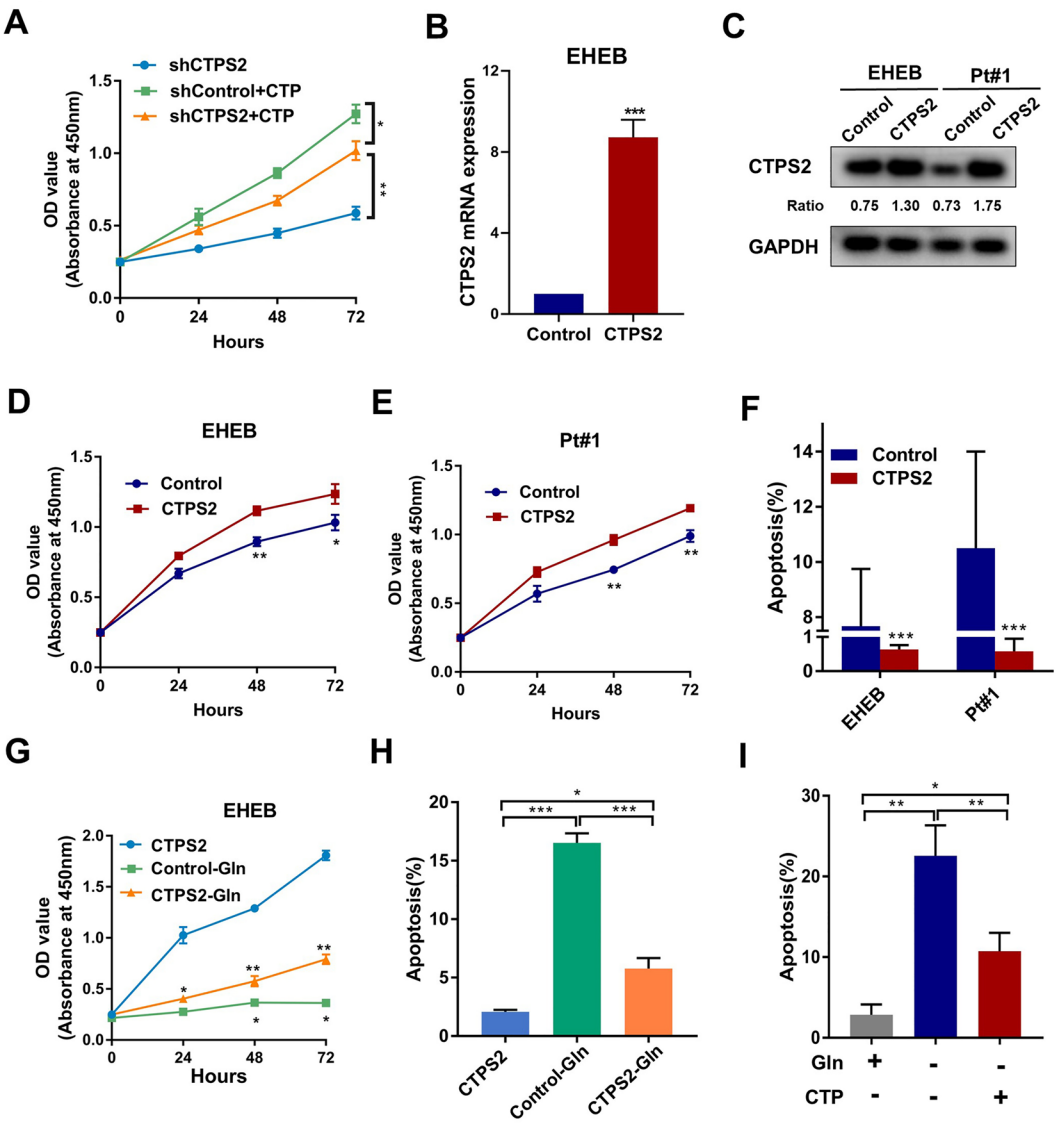
CTPS2 promoted CLL through participating in Gln and CTP metabolism. **A** The addition of exogenous CTP rescued the shCTPS2 induced inhibition of CLL proliferation. **B**, **C** The overexpression efficacy of CTPS2 mRNA and protein was validated in EHEB and primary CLL cells after plasmids transfected. **D**, **E** CTPS2 upregulation enhanced the proliferation of EHEB and CLL primary cells. **F** CTPS2 enforcement impeded the apoptosis of CLL cells. **G**, **H** CTPS2 overexpression could partially rescued the negative effect of Gln-defection on CLL cells’ survival. **I** Comparable rescued effects on apoptosis were achieved by exogenous supplement of CTP under Gln defection. Each experiment was repeated three or more times independently. *p < 0.05, **p < 0.01, ***p < 0.001

To investigate whether CTPS2 promoted CLL progression through affecting CTP synthesis, exogenous CTP was addicted to shCTPS2 and shControl of EHEB cells. As Fig. 4A showed, the addition of CTP significantly rescued the impeded proliferation caused by CTPS2 knockdown. Subsequently, plasmid with CTPS2 overexpression was transfected into EHEB and CLL primary cells, both with effective efficiency (Fig. 4B, C, Additional file 1: Figure S1B). Subsequently, CTPS2 group showed a positive impact on CLL cell proliferation through CCK-8 cytotoxicity assay (Fig. 4D, E). In the Annexin V-PE/7AAD apoptosis assay, CTPS2 enhancement showed a significant attenuation on the percentage of Annexin V + cells (Fig. 4F, Additional file 1: Figure S1C). After successfully establishment of CTPS2-overexpressing cell models, we further detected the effects of Gln and CTP deficiency. EHEB cells with or without CTPS2 overexpression was cultured under Gln-deficient or Gln-rich conditions. Gln-deficient in the media substantially decreased proliferation of EHEB cells with partially recovered by CTPS2 enhancement (Fig. 4G). Meanwhile, CTPS2 upregulated could also alleviated the aberrant apoptosis caused by Gln defection (Fig. 4H, Additional file 1: Figure S1D). Notably, comparable rescued effects were achieved by exogenous CTP addition under Gln defection (Fig. 4I, Additional file 1: Fig. S1E). These data suggested that CTPS2 induced Gln utilization and CTP synthesis to promoted CLL proliferation and survival.

### CTPS2 knockdown attenuated the DNA repair potential in CLL

**Fig. 5:**
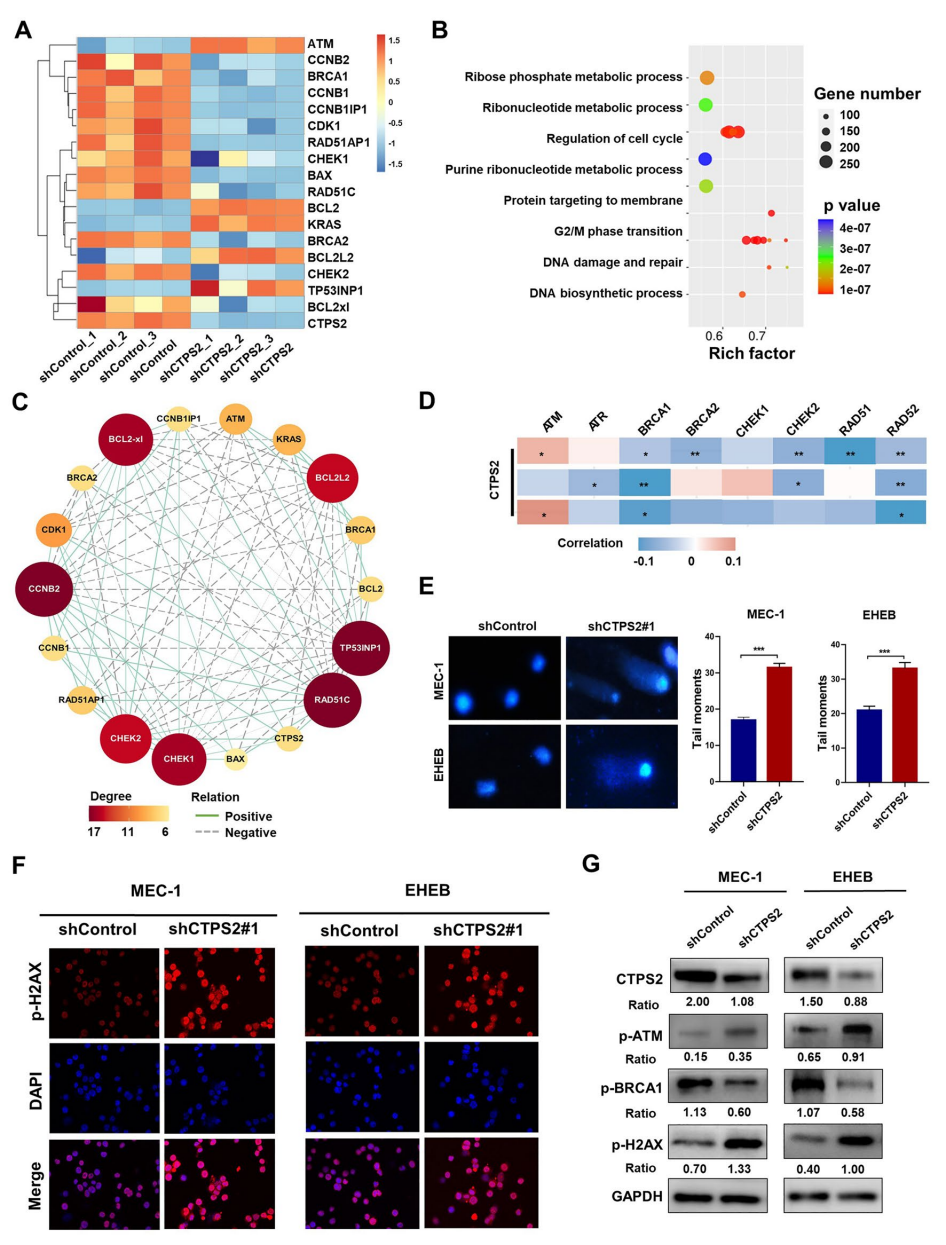
CTPS2 knockdown attenuated DNA repair potential in CLL. **A** The heatmap of differentially expressed (DE) mRNAs detected by RNA-seq in CLL cells with CTPS2 interference. **B**, **C** GO enrichment analysis and correlation network of DE molecules was performed. **D** The correlation analysis of CTPS2 and DDR-related genes. **E** Representative comet assay images of both two cell lines and quantification results. **F** p-H2AX immunofluorescent staining in MEC-1 and EHEB cells after CTPS2 knockdown. **G** CTPS2 silence upregulated phosphorylation of ATM and H2AX and reduced p-BRCA1 in CLL cells. Each experiment was repeated three or more times independently. *p < 0.05, **p < 0.01, ***p < 0.001

To further understand the molecular mechanisms by which CTPS2 enhanced the development and progression of CLL, we further inspected our RNA-seq data. It had been found that 8115 mRNAs were differentially expressed (DE) in shCTPS2 cells, of which 4580 were downregulated and 3535 were upregulated. (Additional file 2: Figure S2A). A heatmap analysis showed that genes involved in apoptosis and DDR signaling were enriched in shCTPS2 (Fig. 5A). Notably, an inverse trend between the RNA-seq results and protein level was observed in Bcl-2 as well as Bax. To validate the RNA-seq data, we conducted additional quantitative PCR experiments. Unlike protein level, no significant alteration was observed in Bax and Bcl-2 mRNA level, suggesting a potential role of CTPS2 in post-transcriptional modification (Additional file 2: Figure S2B). In addition, subsequent pathway enrichment analyses revealed that the DE molecules were mainly related to ribonucleotide metabolic processes, regulation of cell cycle and DDR pathway (Fig. 5B; Additional file 2: Figure S2C). In the DDR-related genes, BRCA2 and BRCA1 showed the greatest change with 0.73-fold and 0.85-fold variation, respectively. Correlation network indicated the interaction between CTPS2 and DDR-related genes, like ATM, RAD51C, Chk1 et al. (Fig. 5C). A further set of correlation analyses was performed in CLL patients from three independent cohorts which showed strong association between CTPS2 and DDR-related regulators (Fig. 5D).

To explore whether CTPS2 can regulate the DDR pathway in CLL cells, the single cell gel electrophoresis or comet assay was performed to estimate overall DNA damage. As Fig. 5E showed, shCTPS2 group displayed longer tail moment, indicating enhanced DNA damage. Moreover, CTPS2 silence induced elevated expression of DNA damage marker (p-H2AX) in CLL cells (Fig. 5F). Subsequently, we examined the phosphorylation of ATM, BRCA1, and H2AX proteins by western blot. As Fig. 5G showed, the shCTPS2 groups were noted to have increased levels of phosphorylated ATM and H2AX, implicating increased levels of DNA damage. Whereas the phosphorylation of BRCA1 (mediate chromosome damage repair by homologous recombination) was decreased, suggesting the alleviated DNA repair potential in shCTPS2 groups. These results indicated that knockdown of CTPS2 was involved in the activation of DDR signaling, thereby beneficial to the DNA damage and reducing DNA repair.

### CTPS2 interacted with BRCA1 in CLL cells

**Fig. 6:**
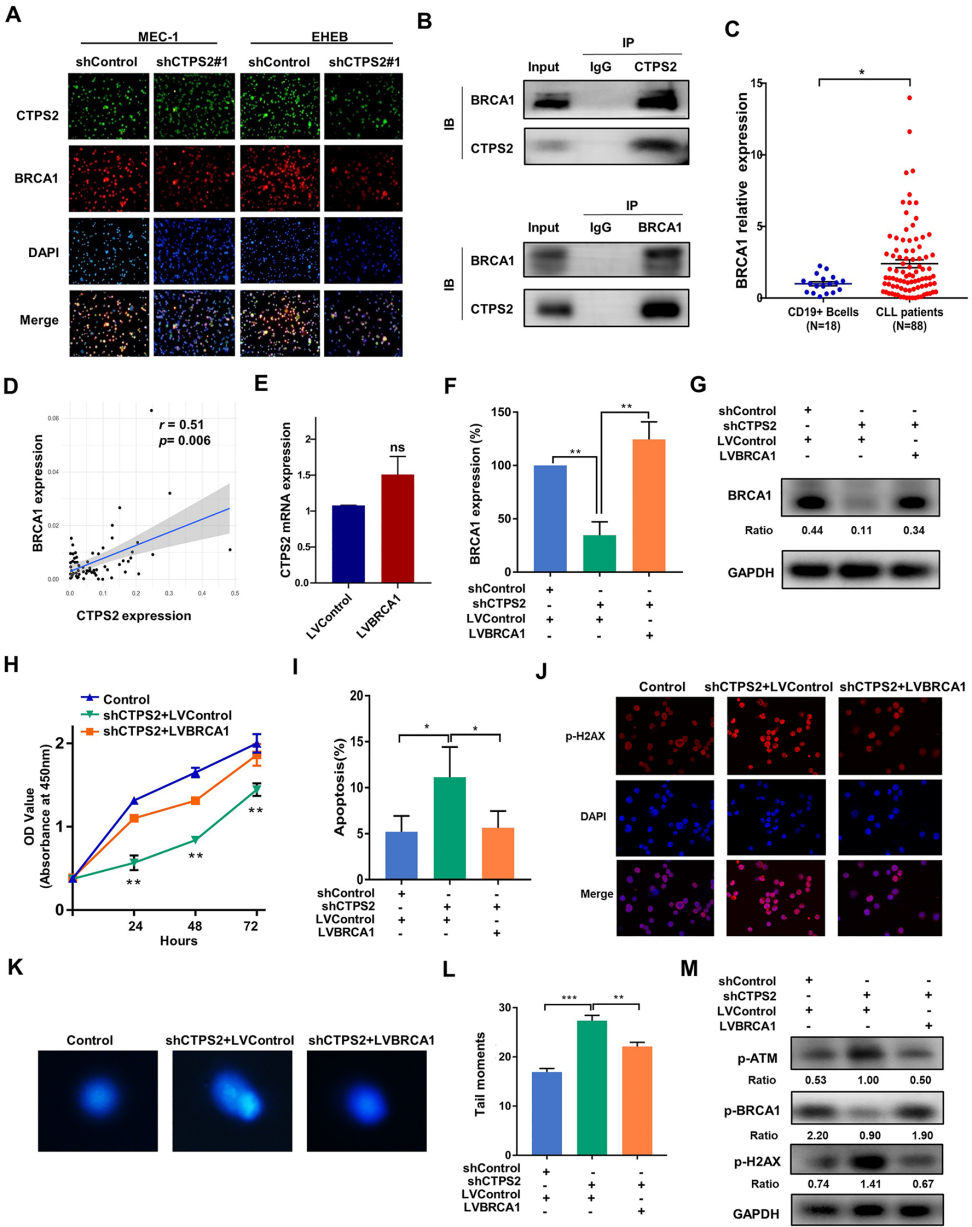
BRCA1 interacted with CTPS2 and rescued the CTPS2 knockdown phenotype in CLL cells. **A** The co-localization of endogenous CTPS2 and BRCA1 proteins was analyzed in MEC-1 and EHEB cells by immunofluorescence microscopy. **B** Co-immunoprecipitation demonstrated that CTPS2 and BRCA1 could be coprecipitated. IgG was used as the negative control. **C** The relative ratio of BRCA1 mRNA in CLL patients versus that in purified normal CD19 + B cells derived from healthy donors. **D** Correlation analysis (Pearson’s correlation) between CTPS2 and BRCA1 expression in CLL patients.** E** The relative expression of CTPS2 in LV-BRCA1 and LV-Control cells. **F**, **G** Validation of BRCA1 expression levels by qPCR and western blot. **H**, **I** BRCA1 overexpression rescued the shCTPS2 induced proliferation defects and apoptosis elevation. **J**–**L** BRCA1 corrected the shCTPS2-induced DNA damage. **M** BRCA1 overcame the downregulation of Bcl-2 and the upregulation of p-ATM as well as p-H2AX after CTPS2 knockdown. Each experiment was repeated three or more times independently. *p < 0.05, **p < 0.01, ***p < 0.001

To clarify the specific association between CTPS2 and the DDR pathway, BRCA1 attracted our particular attention as the significant correlation with CTPS2 among the key components of the DDR pathway. To determine whether CTPS2 and BRCA1 co-localized in CLL cells, we performed immunofluorescence staining with anti-CTPS2 and anti-BRCA1 antibodies in MEC-1 and EHEB cells. The confocal immunofluorescent images illustrated the colocalization of CTPS2 and BRCA1 in CLL cells (Fig. 6A). Meanwhile, a decrease in BRCA1 protein levels in shCTPS2 groups was also corroborated by confocal microscopy. It was observed that endogenous CTPS2 was localized in both the cytoplasm and nucleus, consistent with BRCA1 in MEC-1 and EHEB cells. Subsequently, a co-immunoprecipitation (Co-IP) experiment was further carried out, suggesting the potential interaction between CTPS2 and BRCA1 in CLL cells (Fig. 6B). Analyzing the BRCA1 mRNA expression by qPCR, it was found that BRCA1 was frequently upregulated in CLL patients (Fig. 6C). The expression was also identified to be elevated in the other three cohorts (GSE5006, GSE22529 and GSE55288, Additional file 3: Figure S3A–C). However, the probabilities of overall survival did not differ significantly between BRCA1^high^ and BRCA1^low^ groups in SPHCLL database (Additional file 3: Figure S3D). We further analyzed the prognostic effect of BRCA1 in public data. CLL patients with BRCA1^high^ showed adverse overall survival only in GSE22762 (Additional file 3: Figure S3E). No significance was observed in the larger ICGC data, which was coincided with TTFT (Additional file 3: Figure S3F–H). On the other hand, the expression of BRCA1 showed a close relationship with CTPS2 through correlation analysis (r = 0.51, *p* = 0.006, SPHCLL, Fig. 6D). Subsequently, a lentiviral vector encoding BRCA1 overexpressing (LV-BRCA1) was further constructed and transduced into MEC-1 cells to investigate the role of BRCA1 in CLL (Additional file 3: Figure S3I, J). Overexpression of BRCA1 could accelerate CLL cell growth at 72 h but mildly effect on 24 and 48 h, which may result from the spillover effect (Additional file 3: Figure S3K).

### BRCA1 rescued the CTPS2 knockdown phenotype

**Fig. 7:**
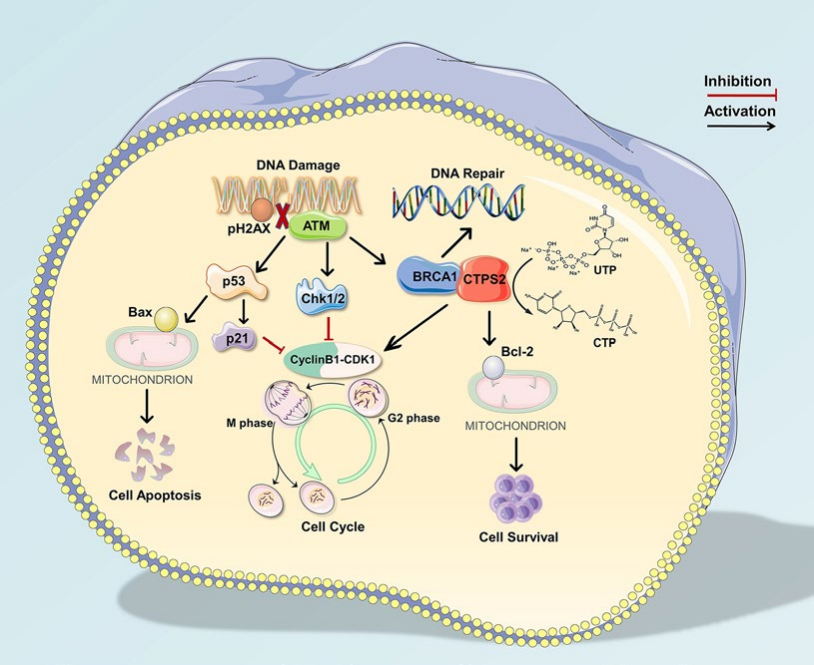
Schematic model of CTPS2/BRCA1-mediated DNA damage response and DNA repair

To understand the regulatory relationship between the two proteins, CTPS2 expression in BRCA1-overexpressed cells was detected by qPCR. BRCA1 enhancement showed no significant effect on CTPS2 expression (Fig. 6E, Additional file 3: Figure S3J), which suggested that BRCA1 functioned as the downstream of CTPS2. To further validate the assumption, we tested whether enhancement of BRCA1 rescued the phenotype associated with CTPS2 knockdown. Stably BRCA1-upregulated lentivirus was transfected into shCTPS2 MEC-1 cells (Fig. 6F, G). Short hairpin (sh) scrambled control and lentivirus vector control were transduced into cells as experimental controls. As expect, the expression of CTPS2 was not affected after additional BRCA1 upregulation (Additional file 3: Figure S3L). It was observed that elevated BRCA1 expression was capable of rescuing the decrease in CLL cell growth (Fig. 6H) and elevation in cell apoptosis causing by CTPS2 knockdown Fig. 6I. Moreover, immunofluorescence staining of p-H2AX (Fig. 6J) and comet assay (Fig. 6K, L) showed that overexpression of BRCA1 rescued CTPS2 shRNA-induced DNA damage elevation. Furthermore, overexpression of BRCA1 overcame the upregulation of p-ATM and p-H2AX after CTPS2 knockdown (Fig. 6M). Taken together, our findings provided supportive evidence that CTPS2 could interact with BRCA1, thereby influencing the biological progress of CLL cells through DDR signaling (Fig. 7).

## Discussion

Our current study provided the first comprehensive analysis of CTPS2 in CLL pathogenesis. CTPS2 was significantly over-expressed in CLL patients, portending a worse overall survival and treatment-free survival. Furthermore, it was demonstrated a precise molecular mechanism that links CTPS2 to the DDR pathway in CLL. Our results indicated that CTPS2 could promote cell survival by reducing DNA damage and increasing DNA repair via interacting with BRCA1.

Prior studies have noted that the activity of CTPS is low in normal tissues but elevated in highly proliferating cells [23]. The cancer cells that exhibited increased cell proliferation also showed increased activity of CTPS2. Weber and co-workers found an elevated CTP synthase activity in hepatoma [15]. Subsequent studies demonstrated that unregulated CTP levels and increased CTP synthase activity are features of many forms of cancer such as leukemia, hepatoma, and breast cancer [24, 25]. Hongwu Fan et al.identified CTPS2 as a critical gene associated with osteosarcoma prognosis [16]. In this study, we unraveled regulatory functions of CTPS2 in CLL by bioinformatic analysis of RNA-sequencing expression profiles, identifying CTPS2 as a crucial prognostic biomarker in CLL patients. For the first time, our data revealed the aberrant expression of CTPS2 in CLL clinical specimens and cell lines. Elevated expression of CTPS2 was significantly correlated with poor overall survival and treatment-free survival of CLL patients, indicating that CTPS2 contributed to the progression of CLL. Down-regulation of CTPS2 retarded CLL cell proliferation mainly by G2/M cell phase arrest and propelling apoptosis in vitro. Notably, the Bax and Bcl-2 expression as verified by qPCR were not consistent with the RNA‐seq results. This inconsistency could be ascribed to a relatively low number of reads for Bax and Bcl-2. Previous studies had reported the ubiquitinated and phosphorylated regulation of CTPS in *Schizosaccharomyces pombe* [26, 27]. These findings suggested a potential involvement of CTPS2 in post-transcriptional modification, which warrants further investigation.

CTPS2 is a critical enzyme that controls the synthesis of cytosine nucleotides and serves a vital role in numerous metabolic processes [28, 29]. CTPS2 has been reported to be involved in several solid tumors, such as osteosarcoma and colorectal cancer, but never in hematological malignancies [16, 30]. The results presented here suggested that the robust activity of CTPS2 in CLL cells could utilize glutamine to support cytidine salvage metabolism and CTP synthesis for DNA replication needs. Following genotoxic damage, cells activate a kinase driven signaling network, referred to DNA damage response and repair [31–33]. Numerous reports had shown that malignant tumors displayed signs of enhanced DNA damage and persistent DDR signaling, likely due to the oncogene-induced replication stress [34, 35]. Ionizing radiation-induced DNA damage was found to accelerate the nucleotide synthesis, resulting in enhanced DNA repair [39]. CTPS2 participated in ribonucleotide metabolic processes by promoting CTP production, suggesting an important role in the DNA replication and repair process. Since the closely clinical relation between the CTPS2 expression and 11q- (ATM gene locus), all of these prompted us to propose that CTPS2 could potentially function in DNA damage and repair. Notably, downregulation of CTPS2 resulted in elevated p-ATM and p-H2AX protein levels coinciding with reduced p-BRCA1 protein levels, which reflected abnormal activation of DDR signaling.

BRCA1 mediated homology-directed repair with other co-factors during DNA replication, thus to protect stressed DNA replication forks [36]. Accumulating investigations have suggested that DDR pathway defects are associated with genomic instability and clonal evolution in CLL [37]. It was reported that combined inactivation of CTPS1 and ATR is synthetic lethal to cancer cells, further highlighting the potential link between CTPS family and DDR signaling [38]. Consistently with the hypothesis, we identified that silencing CTPS2 exhibited anti-leukemia effects and increased the activity of key kinases in the DDR pathway. Furthermore, we illustrated the interaction between CTPS2 and BRCA1 protein through Co-IP assay and immunofluorescence assays. Further experiments proved that BRCA1 could partially rescue the CTPS2 knockdown phenotype as the downstream of CTPS2. Our results provided evidence that the aberrant activation of DDR signaling induced by CTPS2 participated in the regulation of CLL initiation and progression. DDR inhibition showed excellent results in preclinical testing in acute and chronic leukemia [23], which indicated the therapeutic potential of CTPS2 inhibition.

Patients with colorectal cancer accompanied by low CTPS2 expression did not receive a survival benefit from 5-fluorouracil treatment, whereas those with high expression did [30]. Therefore, low CTPS2 expression may be a major determinant for drug resistance. However, the role of CTPS2 in the sensitivity of CLL targeted drugs, such as ibrutinib (Bruton’s tyrosine kinase inhibitor) and venetoclax (Bcl-2 inhibitor), remains unobvious in our work. Further investigation of drugs causing hereditary substance damage will provide more comprehensive conclusions.

Furthermore, the mutation status of IGHV reflected the stage of normal B cell differentiation from which they originate. IGHV mutational status was an important robust individual prognostic marker and related to whether the founder CLL cell clone is of pre- or post-germinal origin [39, 40]. In chronic lymphocytic leukemia, patients with 11q (ATM gene locus) deletion generally displayed a more aggressive clinical course and inferior prognosis in a younger subgroup [41]. However, the physiologic basis of 11q deletions and dysfunction of the ATM gene in the tumorigenesis of hematological malignancies remained unclear. Through the correlation analysis, we found that the overexpression of CTPS2 was associated with 11q deletion and the lack of IGHV mutation, which were indicators of inferior prognosis in CLL patients. And our observations demonstrated that CTPS2 could promote CLL cell survival through DDR pathway, suggesting an underlying molecular mechanism by which ATM functions as a tumor suppressor.

## Conclusions

In summary, it was found that CTPS2 participated in the CLL biological process, functioning as the upstream of BRCA1 to regulate DNA damage and response pathway. Moreover, as the CTP synthetase, CTPS2 could utilize glutamine to support cytidine salvage metabolism and CTP synthesis to promote CLL survival. These results add a fresh perspective to understand the correlation between DDR dysfunction and neoplastic progression. CTPS2 could be extended to other types of malignancies and serves as a nucleotide metabolism target for potential therapeutic intervention for CLL.

## Supplementary Information

**Additional file 1: Figure S1.** CTPS2 induced Gln utilization and CTP synthesis to promote CLL. **A** CTPS2 was upregulated in CLL cell lines, MEC-1 and EHEB cells. **B** The overexpression efficacy of CTPS2 mRNA in primary cells transfected with CTPS2 plasmid. **C **Representative dot plots generated by flow cytometry analysis of CTPS2-overexpression groups versus control. D-E CTPS2 overexpression and CTP addition could both partially elevated the apoptosis of Gln-defection caused in CLL cells. *p<0.05; ***p<0.001.

**Additional file 2: Figure S2.** RNA-seq results of CTPS2 knockdown. **A** Volcano plots showing differentially expressed genes. **B** The expression level of Bax and Bcl-2 was validated through qPCR assays.** C **KEGG enrichment analysis of differentially expressed transcripts.

**Additional file 3: Figure S3.** BRCA1 overexpression promoted cell proliferation.** A-C** BRCA1 was markedly upregulated in CLL public database by silico analysis. Analysis was based on GSE5006, GSE22529 and GSE55288, respectively. **D-F** Kaplan-Meier curves of overall survival in BRCA1^low^ and BRCA1^high^ groups. Analysis was based on SPHCLL, GSE22672 and ICGC cohorts, respectively. **G-H** BRCA1 expression did not affect time to first-treatment in CLL. Analysis was based on GSE22762 and GSE39671, respectively. **I-J** mRNA and protein expression of the BRCA1 was assessed by qPCR and western blot after transfecting. **K** Overexpression of BRCA1 amplified the cell growth in CLL cells. **L** The level of CTPS2 expression was not affected with additional BRCA1.

## Data Availability

The datasets supporting the conclusions of this article are available in the GEO database (http://www.ncbi.nlm.nih.gov/geo/) and the International Cancer Genome Consortium database (https://icgc.org/). The other data and materials are available from the corresponding authors upon request.

## Abbreviations

CLL: Chronic lymphocytic leukemia
CTPS2: Cytidine triphosphate synthase 2
OS: Overall survival
ROC: Receiver operating characteristic curves
DDR: DNA damage and response
Co-IP: Coimmunoprecipitation
