# Supplementary figures and images for: CTP synthase 2 predicts inferior survival and mediates DNA damage response via interacting with BRCA1 in chronic lymphocytic leukemia
Source: Exp Hematol Oncol. 2023 Jan 12;12:6. doi: 10.1186/s40164-022-00364-0 (PMC9835321; doi:10.1186/s40164-022-00364-0)

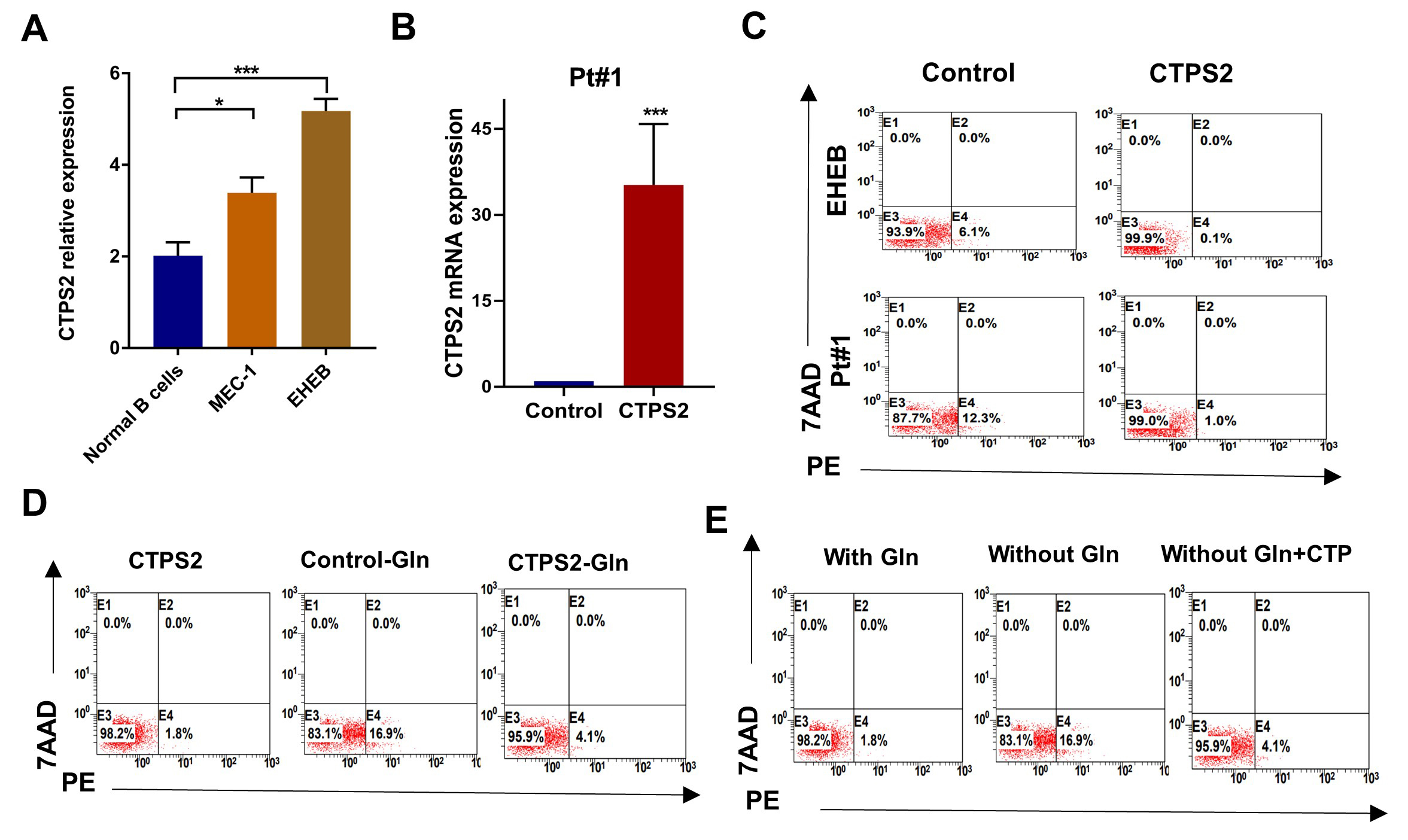

Supplement: Supplementary file 1 — Additional file 1: Figure S1. CTPS2 induced Gln utilization and CTP synthesis to promote CLL. A CTPS2 was upregulated in CLL cell lines, MEC-1 and EHEB cells. B The overexpression efficacy of CTPS2 mRNA in primary cells transfected with CTPS2 plasmid. C Representative dot plots generated by flow cytometry analysis of CTPS2-overexpression groups versus control. D-E CTPS2 overexpression and CTP addition could both partially elevated the apoptosis of Gln-defection caused in CLL cells. *p<0.05; ***p<0.001. [file 40164_2022_364_MOESM1_ESM.jpg]

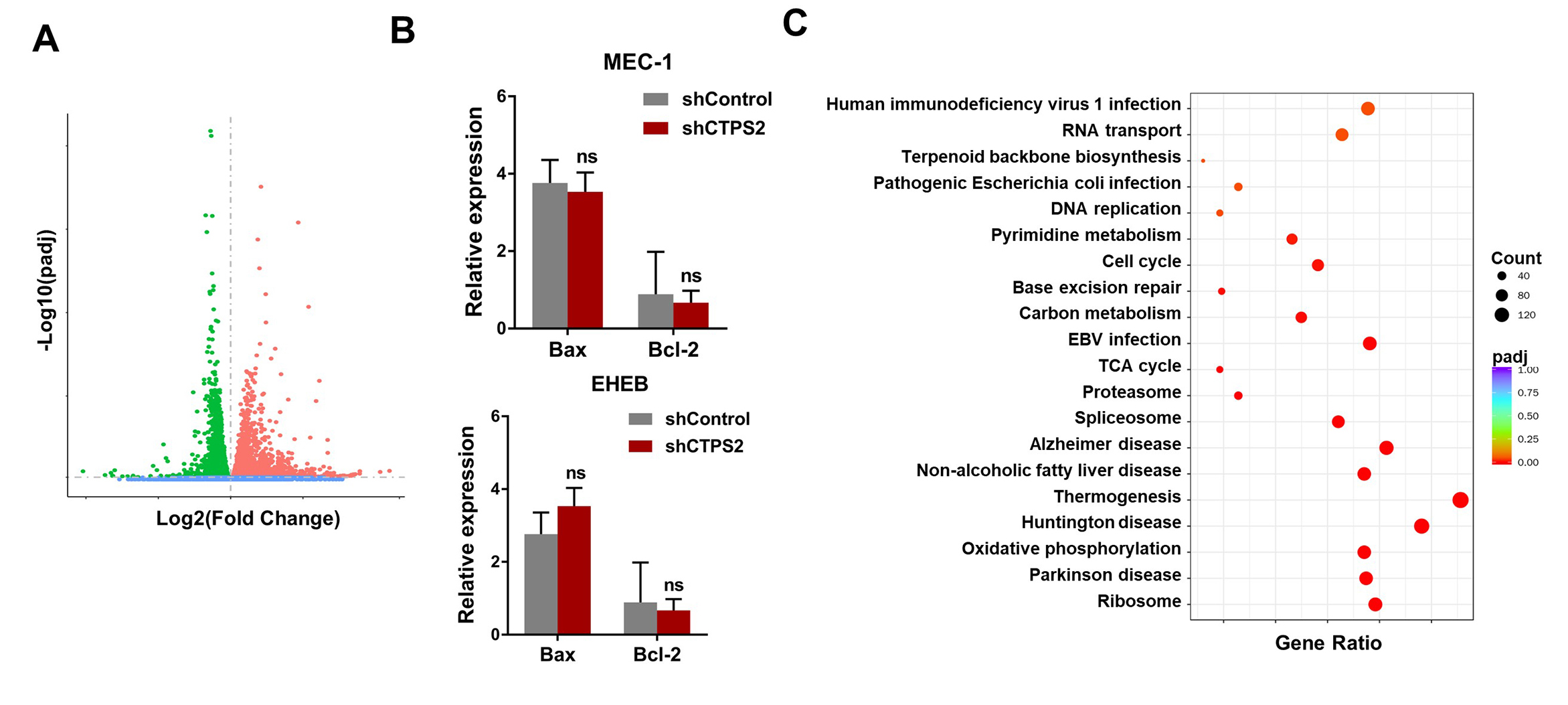

Supplement: Supplementary file 2 — Additional file 2: Figure S2. RNA-seq results of CTPS2 knockdown. A Volcano plots showing differentially expressed genes. B The expression level of Bax and Bcl-2 was validated through qPCR assays. C KEGG enrichment analysis of differentially expressed transcripts. [file 40164_2022_364_MOESM2_ESM.jpg]

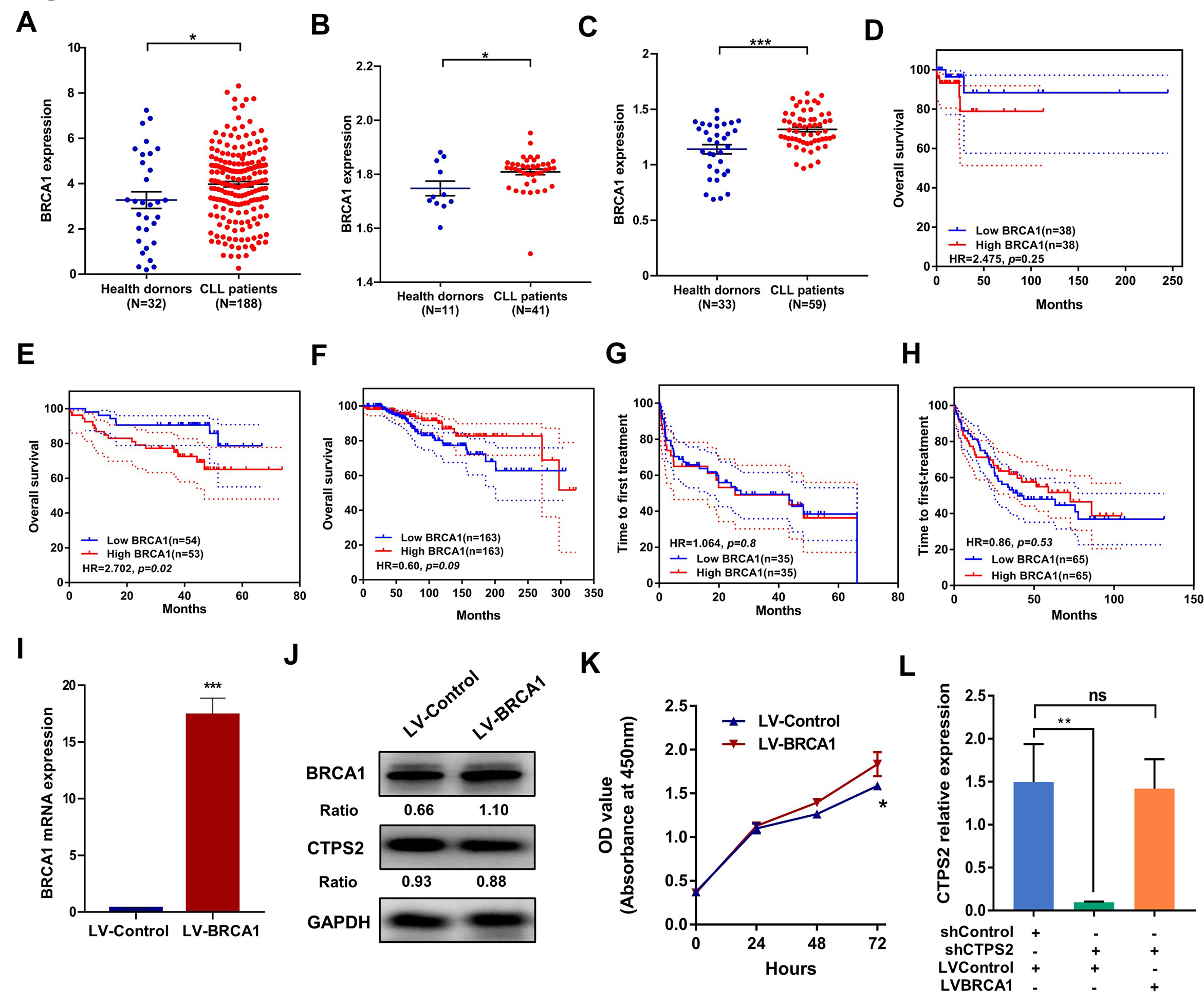

Supplement: Supplementary file 3 — Additional file 3: Figure S3. BRCA1 overexpression promoted cell proliferation. A-C BRCA1 was markedly upregulated in CLL public database by silico analysis. Analysis was based on GSE5006, GSE22529 and GSE55288, respectively. D-F Kaplan-Meier curves of overall survival in BRCA1low and BRCA1high groups. Analysis was based on SPHCLL, GSE22672 and ICGC cohorts, respectively. G-H BRCA1 expression did not affect time to first-treatment in CLL. Analysis was based on GSE22762 and GSE39671, respectively. I-J mRNA and protein expression of the BRCA1 was assessed by qPCR and western blot after transfecting. K Overexpression of BRCA1 amplified the cell growth in CLL cells. L The level of CTPS2 expression was not affected with additional BRCA1. [file 40164_2022_364_MOESM3_ESM.jpg]
